# No Pet or Their Person Left Behind: Increasing the Disaster Resilience of Vulnerable Groups through Animal Attachment, Activities and Networks

**DOI:** 10.3390/ani4020214

**Published:** 2014-05-07

**Authors:** Kirrilly Thompson, Danielle Every, Sophia Rainbird, Victoria Cornell, Bradley Smith, Joshua Trigg

**Affiliations:** 1 Appleton Institute, Central Queensland University, 44 Greenhill Road, Wayville, Adelaide, SA 5034, Australia; E-Mails: d.every@cqu.edu.au (D.E.); s.rainbird@cqu.edu.au (S.R.); b.p.smith@cqu.edu.au (B.S.); j.trigg@cqu.edu.au (J.T.); 2 Faculty of Medicine, Nursing and Health Sciences, Flinders University, Adelaide, SA 5001, Australia; E-Mail: victoria.cornell@flinders.edu.au

**Keywords:** pets, animal attachment, natural disasters, vulnerability, resilience, protective factors, risk factors, preparedness, response and recovery

## Abstract

**Simple Summary:**

The potential for reconfiguring pet ownership from a risk factor to a protective factor for natural disaster survival has been recently proposed. But how might this resilience-building proposition apply to members of the community who are already considered vulnerable? This article addresses this important question by synthesizing information about what makes seven particular groups vulnerable, the challenges to increasing their resilience and how animals figure in their lives. It concludes that animal attachment could provide a novel conduit for accessing, communicating with and motivating vulnerable people to engage in resilience building behaviors that promote survival and facilitate recovery.

**Abstract:**

Increased vulnerability to natural disasters has been associated with particular groups in the community. This includes those who are considered *de facto* vulnerable (children, older people, those with disabilities *etc*.) and those who own pets (not to mention pets themselves). The potential for reconfiguring pet ownership from a risk factor to a protective factor for natural disaster survival has been recently proposed. But how might this resilience-building proposition apply to vulnerable members of the community who own pets or other animals? This article addresses this important question by synthesizing information about what makes particular groups vulnerable, the challenges to increasing their resilience and how animals figure in their lives. Despite different vulnerabilities, animals were found to be important to the disaster resilience of seven vulnerable groups in Australia. Animal attachment and animal-related activities and networks are identified as underexplored devices for disseminating or ‘piggybacking’ disaster-related information and engaging vulnerable people in resilience building behaviors (in addition to including animals in disaster planning initiatives in general). Animals may provide the kind of innovative approach required to overcome the challenges in accessing and engaging vulnerable groups. As the survival of humans and animals are so often intertwined, the benefits of increasing the resilience of vulnerable communities through animal attachment is twofold: human and animal lives can be saved together.

## 1. Introduction

Humans form strong attachments to pets and other animals. The complex interdependency of human and animal survival has been demonstrated during natural disasters when people risk their own lives to save those of animals [1,2] or lose their lives trying. However, rather than see animal attachment as a risk factor for human survival, there is significant potential for using animal-attachment to motivate disaster preparedness, early evacuation and survival [3]. That is, animal attachment can be used to motivate actions that improve survival and increase disaster resilience. However, resilience is not evenly distributed across the community [4] (p. 2), leaving some groups more vulnerable than others. The Australian National Strategy for Disaster Resilience [4] warns that the level of exposure to disaster risk, understanding of risk, and ability to respond and recover from disasters vary. Factors such as remoteness, mobility, age and speaking English as a second language can all play a role in the vulnerability of individuals to disasters. As animal attachment can be seen as an additional factor, there is a need to understand how animals and pet ownership impact the disaster resilience of vulnerable people.

In Australia, five groups are commonly described as ‘vulnerable’: Indigenous Australians, culturally and linguistically diverse communities (CALD), children and youth, the elderly and people with disabilities [5]. Two other groups also warrant consideration: the homeless and people with mental health issues. The difficulties in engaging vulnerable groups in disaster resilience initiatives include access, resonance, motivation, communication and language. That is, how do we find them, why should they care, what would make them act, how can we contact them and what form should our communications, warnings and messaging take? As vulnerable people can often be socially isolated and not always literate, innovative engagement strategies are required. Engaging vulnerable people in disaster resilience strategies through their desire to save pets and animals could be one effective approach. We use the term ‘vulnerable’ as a term of convenience and imply that these groups are under a higher level of risk (or ‘at risk’), but appreciate that not all members within a group have the same potential to be resilient and resourceful.

As discussed below, pets and animals are important to vulnerable people for practical and personal reasons. It follows that pets and animals impact the emergency behavior and disaster resilience of those vulnerable people who own or care for them. For instance, vulnerable people may be reluctant to evacuate without their animals, may decline emergency accommodation if their animal is unwelcome, and may struggle more than others to cope with recovery and rebuilding without them. This article suggests that animal attachment and animal-related activities could be successful vehicles for disseminating information and engaging vulnerable people in strategies designed to increase resilience. As argued in relation to the general population [3], encouraging vulnerable groups to think about emergency planning for their pet or animal could motivate them to prepare for themselves. In addition, where there is a need for an innovative approach that resonates strongly with a target audience—as is the case with vulnerable groups—animal attachment could be particularly effective in engaging animal owners and guardians in disaster resilience behaviors. That is, pet and animal-related activities and social networks could be used as conduits for disseminating disaster resilience information and engaging pet and animal owners in disaster resilience building behaviors.

This success of ‘piggybacking’ one message onto another can be seen in the promotion of dog walking as a public health strategy to increase human activity [6]. Such opportunistic messaging also underpins comments in the guide to Vulnerable Persons Registers. For example,

*If someone calls for themselves or a family member or friend, this is a good opportunity to provide someone with information about emergency planning resources and to encourage them to undertake some planning activities or to prompt the person they are calling about to connect with local community or personal networks*.

[7]

Similarly, incorporating disaster resilience materials and strategies in existing animal-related motivations, activities and social networks could serve to increase disaster awareness, preparedness and resilience. Indeed, resilience is linked to a community’s ability to ‘use local networks and resources to support actions required during an emergency and to support recovery efforts’ [4] (p. 10). As such, this article supports the earlier assertion that animal attachment could be used to increase disaster resilience and survival across the general population [3]. However, animals are arguably more important to people for whom the challenges associated with disaster resilience are compounded by vulnerabilities.

## 2. Method

Before considering the relationship between animals and disaster resilience, it is necessary to provide a note on the material that supports this paper. The authors searched English language academic literature from October 2013 to December 2013 with a focus on the seven vulnerable groups: Indigenous Australians, culturally and linguistically diverse communities (CALD), children and youth, the elderly, people with disabilities, the homeless and people with mental health issues. Given the paucity of available literature on the topic of improving the resilience of specific vulnerable communities using animal attachment, and given the diversity of the groups, there was no systematic procedure followed, and no time limit on academic literature. However, by synthesizing literature on what makes these groups vulnerable, the challenges to building their disaster resilience and how animals figure in their lives, it was possible to develop some literature-informed propositions.

Notwithstanding the fact that general emergency management for all seven groups is under-researched, this article is the first to synthesize information on vulnerability, disaster resilience and pet ownership across the groups to concurrently consider the topic of animals, disasters and vulnerability. Whilst the majority of research has been focussed on domestic pets such as cats and dogs, this article is interested in animals broadly (pets, companion animals, livestock, wildlife).

## 3. What Do Animals Have to Do with Disaster Resilience?

Humans form close interpersonal relationships that are commonly referred to as ‘attachments.’ Within psychology, Attachment Theory is associated with John Bowlby [8] and Mary Ainsworth’s [9] research on affectional ties between a primary caregiver and infant. It has since been extended to describe the close bonds formed between humans and non-human animals [10,11,12]. The essential premise of Attachment Theory is that individuals exercise attachment strategies, with the goal of establishing and maintaining proximity to an attachment figure’ [8]. To act as a source of psychological security, attachment figures should provide:

(1) Proximity maintenance—they are sought out and available in times of need;
(2) Safe haven—they offer protection and support to relieve distress;
(3) Secure base—they act as a reliable presence that facilitates and permits risk-taking and exploration; and
(4) Separation distress—prompted by separation from or actual loss of the figure [13].

The threat of actual or symbolic danger to the relationship triggers attachment strategies that employ these criteria seen, for example, in the seeking of proximity to the figure (or the reluctance to abandon an animal).

Research suggests that companion animals—particularly dogs—satisfy the above criteria, with proximity maintenance [14], as well as safe haven and secure base [15] the most salient features. According to Ainsworth, this overall perception of felt security and comfort is a fundamental characteristic of the attachment-type affectional bond to a unique figure that ‘… is never wholly interchangeable with, or replaceable by another …’ [16] (p. 711). Many guardians would agree that their animal charges are unique and irreplaceable. Attachment Theory then represents a valuable tool for understanding why people risk their lives to save animals during natural disasters. However, the willingness of people to save animals such as wildlife with whom they do not demonstrate a ‘textbook’ attachment relationship suggests that other explanations may be required to explain the willingness of humans to save animal lives; that this behavior is driven by other motivations besides attachment (such as obligation and morality); or that attachment could manifest in more spontaneous and transient forms, such as hope symbolism [17]. Whatever the motivation, such actions are open to public interpretation and re-interpretation, mostly according to the outcome of animal rescue attempts [18].

Regardless of the terminology (for a discussion of ‘animal as extended self’ [19,20] as an alternative theory to explain human risk-taking behavior to save animals, see Thompson [3], there is clear evidence that the safety of humans and animals is intertwined during disasters [1,2,21]. The literature on animals and disasters is limited, yet diverse. It spans the impact of pet ownership on evacuation failure [1,2], the anthropogenic causes of mass animal deaths during natural disasters—especially in relation to intense animal farming or production [21], emergency accommodation for animals, public health implications of abandoned pets and pets in shelters, implications for responders and emergency services personnel [22,23], implications for animal rights advocates [24], the planning, management and administration of animals during disasters [21,25,26], the emotional impact of pet loss following disasters [23,27], risk factors for pet evacuation failure [1], and risk factors for losing a pet during a disaster [28]. Together, this body of research characterizes animal ownership as a risk factor for disaster survival.

Indeed, over 8% of flood-related fatalities in Australia from 1788 to September 1996 resulted from people’s attempts to save ‘stock, property or pets’—even when the animal or pet was not their own [29] (p. 406), such as when ‘good Samaritans’ attempted animal rescue. Further afield, Heath, Kass and others found that Californian animal owners were less likely to evacuate than non-owners [2]. Their study on flood evacuation behavior found ownership of pets the greatest reason for failure to evacuate in houses without children, with the risk of failing to evacuate increasing twofold for every additional companion cat or dog in the household [2]. Howlett and Turnbull cite a USA study finding that more than 80% of animal owners would risk their lives to save their animal [30] (p. 3). Heath, Voeks *et al.* identified that when animal owners do evacuate, they are likely to return to try to rescue their animals [1]. When people risk their lives to save or return to animals, they also endanger the lives of others [24] such as family, friends, neighbors, rescue and relief personnel.

However, given that more than half the population own pets, there is arguably more risk in **not** helping people to safely accommodate animals in their emergency plans. There are high levels of pet ownership in developed societies, such as 63% in Australia and the US [26,31]. That leaves another large percentage of the population with animals in their lives, through co-location or their social relations with pet owners. In fact, pet ownership and animal interactions are an important part of everyday life in Australia, with 91% of Australian pet owners reporting feeling ‘very close’ to their pet [32] (p. 73). Shifting focus from the risk of animal ownership to the risk of not helping animal owners/guardians to save themselves and their animals might just save more lives. That is, the idea of animal attachment as risk factor could be reconfigured to one of protective factor [3]. By motivating people to develop and activate their emergency preparedness and response [26], animal attachment could improve the survival rates of animal owners, guardians and others who may risk their lives to rescue animals such as the ‘good Samaritans’ mentioned above in relation to flood-related deaths.

Animals are no less significant in the recovery and rebuilding phases of a disaster, due to the psychological impact of animal loss [33,34]. Whilst the emotional impact of the loss of human life is widely acknowledged, the loss of animals can also result in significant grief and psychological trauma [27]. When animal loss occurs alongside a traumatic event such as a disaster, the impact can be overwhelming [28]. In the case of a natural disaster, humans often experience ‘post-disaster distress’ [27], especially following ‘enforced abandonment’ [35] of animals or feelings of blame for not having made the necessary precautions for the life of their animal. They may also experience guilt because their grief of losing an animal is socio-culturally less valued than the grief of losing a human. This trauma is not specific to close relations with domestic companion animals. Farmers can also experience psychological trauma from the loss of livestock and substantial financial losses [23]. For example, the biological disaster of an outbreak of foot and mouth disease in the UK in 2001 which involved the slaughter of more than four million cows, pigs and sheep also resulted in more than 80 suicides by farmers and other affected people [21] (pp. 14–15). Therefore, helping people to save animals is relevant not only to disaster planning and survival but to recovery and rebuilding in the days, weeks, months and years after a disaster.

In the same way that animal attachment could be reconfigured from risk factor to protective factor for natural disaster survival, so too could vulnerability be converted into resilience. The term resilience is widely used across many disciplines, for example ecology, engineering, and psychology; each conveying their own nuance. However, ‘...all definitions are linked to the ability of a system, entity, community or person to withstand shocks while still maintaining its essential functions’ [36] (p. 2). Of particular relevance to this article, is community resilience. Community resilience can be complex, as ‘it involves the interaction of individuals, families, groups and the environment’ [37] (p. 9) and it ‘is dependent on social interaction and collective action based on networks of relationships, reciprocity, trust, and social norms’ [37] (p. 11). A resilient community is one whose members are connected to one another and work together, and which can adapt to changes in the physical, social or economic environment. The role that animals can play in community connectedness is revealed in research on pets and social capital [38]. That is, pets provide social capital through which people can interact with others, build social networks and feel good about their local neighborhood [39]. In this way, they can counter social isolation that has been linked with vulnerability.

The Victorian Vulnerable People in Emergencies Policy defines a vulnerable person as ‘someone living in the community who is: frail, and/or physically or cognitively impaired; and unable to comprehend warnings and directions and/or respond in an emergency situation’ [40] (p.3). For inclusion on the state Vulnerable Persons Register, a person needs to additionally be unable to ‘identify personal or community support networks to help them in an emergency’ [40] (p. 3). Despite the accepted view that pets provide social capital [39], that social capital is important to resilience [41], and that animals provide psychological, emotional, health, wellbeing and practical benefits to vulnerable people [42], there has been no systematic investigation of the impact of animals on the resilience of vulnerable groups. This article is the first to consider the ways in which animals could be used to increase the disaster resilience of vulnerable groups. They are discussed below, according to their characteristics and prevalence, challenges to their disaster resilience, the role of animals in their lives and the impact of animals on their resilience. Once these aspects are outlined, we hypothesize the potential value of using animal attachment to improve the resilience of these vulnerable groups.

## 4. Vulnerable Groups and the Roles of Animals in Their Lives

To determine the extent to which animal attachment could be used to increase the disaster resilience of vulnerable groups in the community, it is important to first understand who those groups are, what makes them vulnerable to disasters, and how animals play a role in their lives. Seven vulnerable groups are discussed below, in no particular order.

### 4.1. Indigenous Australians Living in Remote Communities

The term ‘Indigenous Australian’ refers to those who identify as being of Aboriginal or Torres Strait Islander origin, and who are accepted as such by the community with which the persons associate [43]. There is a growing movement of Indigenous Australians away from major centers to outstations and homeland communities. This has led to the establishment of small Indigenous settlements in often extremely remote locations [44,45]. The development of remote communities has been identified as a particular challenge for disaster resilience [4] (p. 12) because they are typically located in areas that are susceptible to natural hazards such as cyclones, flooding and bushfire. These communities can be difficult to relocate during natural disasters for reasons of tradition, culture and cost. Communities are often forced to seek shelter or refuge in inappropriate buildings, or forced to evacuate by air due to damage caused to access roads.

Animals feature heavily in the lives of Indigenous lifestyle and spirituality [46]. Dogs are the most common companion animals found in Indigenous communities as they are considered an intrinsic part of the community. Unfortunately, pet ownership numbers are not reported for Indigenous peoples. However, it is not uncommon for domestic dogs to outnumber people in Indigenous communities [47,48]. Indigenous Australians have an enduring relationship with dogs that first began with dingoes [49]. The relationship between dogs and humans is intricately linked, and this relationship has changed over time incorporating new perspectives and dog keeping practices. Both dingoes, and now dogs, are considered sacred animals and deeply incorporated into Aboriginal society. For example, they are formally included in family units, (*i.e.*, they are afforded traditional or kinship names) and incorporated into creation and dreaming knowledge [50]. Indigenous people in contemporary society own dogs for a number of reasons, including companionship, as a physical and spiritual protector, to assist in hunting, and as a source of warmth [51].

There are three categories of dogs found in Indigenous communities; (1) the traditionally valued hunting dogs, (2) the community or free-living community dogs, and (3) the pet dogs. Hunting dogs are named and incorporated into the local kinship system. They are highly valued, play an important role in protecting and feeding the community and are often purchased and/or brought in from outside the community. Community dogs are typically unnamed and loosely attached to households, but not directly owned by anyone. These dogs usually fend for themselves and obtain food through eating disregarded remains or fighting favored dogs (hunting dogs, or those that are owned and directly cared for). Another is the pet dog. These are given names, collars, allowed in the house, and generally well fed and cared for [47].

Regardless of a lack of formal ownership and direct care, community dogs are still highly meaningful and useful to the community [46]. For example, around households the dogs combine with the other dogs to create a body of animals protecting a household from both human and spirit intruders [47]. On a community level, larger dog populations are perceived as having special intuitive powers. It is believed that dogs sense human deaths and usually alert the community of a death by howling [47]. Although many of the dogs in communities appear neglected to outsiders [46], this is not entirely reflective of care and ownership, but more a lack of knowledge and/or access to veterinary care.

In relation to disaster response, if evacuation is essential, it is important to note that there may be some individual dogs that are more likely to influence decision to evacuate. For example, companions and prized hunting dogs are likely to be prioritized for evacuation with people, as they will play an important functional role in the aftermath of a disaster for protection and hunting for food. However, this is not to downplay the important social, cultural and practical function of all dogs in remote Indigenous Communities and other animals. Many Indigenous people also share a special relationship with some natural species, or a particular member of that species (e.g., a totem animal such as a kangaroo, eagle *etc.* [50]). Some may choose to stay to protect or care for these animals instead of evacuating.

Evacuations from remote areas are often conducted by air and residents of the community temporarily relocated, so the evacuation of companion animals is problematic. In one published case study, the entire remote community of Kiwirrkurra, Western Australia (170 people) was evacuated to a military base in 2001 due to flooding. This represented a severe disruption to the social fabric of the community, and members commented that their separation contributed to social difficulties. During interviews, residents spoke specifically about how devastated they were to leave their dogs behind. To them, dogs were (and remain) an important cultural element of the community [52]. This suggests that domestic dogs are likely to be an important factor in successful recovery in remote Indigenous communities.

### 4.2. Culturally and Linguistically Diverse Communities

Like many countries, Australia’s population is culturally and linguistically diverse (CALD). CALD people may be migrants, refugees, asylum seekers, international students or second or third generation Australians. 30.2% of Australia’s population was born overseas, and of the 69.8% of the population who were born in Australia, 34.3% had both parents born overseas and 11.9% had one parent born overseas [53]. CALD people bring with them a rich array of cultures and languages. A language other than English is spoken at home by almost one quarter (23.2%) of all Australians. Indeed, CALD people may speak one of some 50 languages other than English as recorded by the Australian Bureau of Statistics, including Mandarin (1.6%), Cantonese (1.2%) and Greek (1.2%) [53].

CALD people are more vulnerable to disasters because they may have limited access to information in a relevant language. Following hurricanes and flooding in the US, numerous studies found that ethnic minorities, particularly those with limited English language skills, were hit hardest [54,55,56,57]. Following Hurricane Katrina for example, many CALD residents did not evacuate ‘because the government failed to issue warnings, evacuation instructions, or hazard and safety precautions in a language they could understand’ [58]. Although translation of these materials is typically offered as a solution, low education attainment can create further challenges for CALD people that are not always resolved by good translation. For example, it is possible that some refugees from South Sudanese may be illiterate in their own language [59]. Moreover, information cannot simply be translated, but needs to be culturally appropriate. Important messages can be lost in literal or cultural translation [58,60,61]. Although CALD people are considered to be vulnerable to hazards and risks associated with disasters [58], those from refugee backgrounds in particular are likely to draw on their experience and skill in surviving adversity [62].

The limited literature on pet ownership amongst CALD communities is largely US-based. For instance, studies have found that ‘white’ people are more likely to own pets than Hispanics, African Americans or Asians [63,64,65]. However, the term ‘white’ is not defined and does not take into consideration the cultural and linguistic diversity that ‘white’ may encompass. Hood’s [66] research on pet ownership amongst Asians in Australia makes a similar point; it is equally important to recognize ‘cultural, religious and ethnic differences’ in pet ownership amongst Asian communities as it is to avoid the mistake of applying ‘unhelpful generalizations and stereotyping’ [66].

There are some aspects of pet ownership that are unique and specific to particular CALD cultural and religious groups. Dogs might be seen as unclean by some followers of Islam (in accordance with the Qur’an), and are unlikely to be kept as pets in homes [67]. Consequently, in an emergency shelter scenario, some individuals may not appreciate a dog being present [66]. Walker, Robinson and others [68] found that members of CALD communities were less concerned with the ‘fate of pets’ than non-CALD (cited in [69]). However, only three of the CALD participants in that particular study had responsibility for pets. Whilst CALD pet ownership levels appear low, they may increase across generations. Any benefits of using animal attachment to increase disaster resilience amongst vulnerable communities should thus not be precluded from CALD communities on the assumption of low ownership levels alone.

### 4.3. Children and Youth

UNESCO identifies children as those under 18 years of age, and youth as those aged between 15 and 24 years [70]. Children and youth equate to 33% of Australia’s population [71]. There is a wealth of literature referring to the psychological effects of disaster on children and youth [72,73,74,75,76,77]. The degree of psychological symptom development is proportionate to the extent of close-exposure of the disaster experienced by the child [78]. Despite young people being referred to as one of the groups most vulnerable to natural disasters [79], school-age children have been found to lack the emergency management training that could assist them and their families to be more resilient [80]. Indeed, Mitchell *et al.* argue that too much emphasis has been placed by researchers (citing [81,82]) on children’s vulnerability as ‘passive victims with no role to play in communicating risk and participating in decision-making processes’ [83]. Instead, they argue that because of the high toll of child deaths in disasters, children should be involved in disaster planning.

According to Chaseling [84], pet ownership is highest amongst families with children. Dogs and cats are the most popular pets, whilst there are also high numbers of pet birds and fish [84]. Research has found that having a pet improves children’s health outcomes [85,86]. Animals improve the lives of children with disabilities, for instance Assistance Dogs Australia supporting children with autism [87,88,89]. Pets are also understood to ‘aid childhood development especially nurturing and social skills’ [90]. They can be particularly important in helping children who are experiencing stress [91]. Indeed, studies have found benefits of animals including a decrease in stress including anxiety and fear in hospitalized children when receiving Animal-Assisted Therapy [92]. Of particular importance to the role that animals can play in assisting children to cope with and recover from a disasters, pets ‘improve feelings of safety and help create social bridges in our communities’ [90]. According to Ross *et al.* [93], children may ‘view their pet as they would a human sibling’ or as a best friend, confidante, ‘source of comfort and protection’ [93] (p. 81).

In a study of the extent of evacuation during a flooding disaster in the US, families with children were more likely to evacuate than those without children. The study also found that ‘pet ownership is associated with household evacuation failure’ [2]. The concern is that a family’s likelihood of evacuating decreases with pet ownership. Indeed, in an earlier study, Heath *et al.* [94] found that families with children were most likely to re-enter an evacuated area to rescue their pets. However, the risk to children here is not so much in pet ownership, as it is in failing to ensure that children and pets remain safely together during a disaster event. According to Travis [95], pets have a huge impact on the disaster resilience of children, because of the stability pets provide during a traumatic and disruptive event. Research has found that the death of a pet can have a profound effect on a person’s emotional health [96,97]. Indeed, the forced separation of a pet during an evacuation can produce the same grief reaction as would the death of a family member or close friend [98]. The most renowned example of a forced separation was witnessed by people around the world as footage of the Hurricane Katrina evacuations recorded a dog named Snowball being torn from the arms of a distraught young boy who was not allowed to bring his pet on a bus [99].

### 4.4. Older People

Most developed world countries have accepted the chronological ages of either 60 or 65 years as a definition of an older person, which in many cases aligns with the age at which a person becomes eligible for statutory and occupational retirement pensions [100]. As many disaster researchers have highlighted (for example, [101]), it is not advancing age alone that makes older people vulnerable. Rather, age is accompanied by certain characteristics that may decrease resilience:

*In addition to chronic health conditions, older adults may have impaired physical mobility or cognitive ability, diminished sensory awareness, and social and economic limitations. For example, declining vision or hearing can make it difficult for an older adult to communicate. Older adults with cognitive problems may become agitated during a crisis or feel overwhelmed by the crowding, noise, and lack of privacy in a shelter. They may need assistance to ensure that they have their medications, adequate nutrition and water, and assistive devices*.

[102] (p. 2)

Older people are sometimes considered to be a challenge with respect to disaster planning due to their perceived reluctance to prepare and evacuate [103,104,105]. Following disasters, older people often suffer financial hardship and social isolation, exacerbation of existing health conditions and exposure to new health conditions (linked to loss of social support and assistive aids and to impaired access to health services) [106].

As with other vulnerable groups, the current level of animal ownership amongst older Australians is not well understood. In 1994, 32.3% of lone persons aged 60 years or over had a pet [107]. This level is not necessarily indicative of the significance of pets and other animals to older people, and some older people may actively choose to not have pets. Chur-Hansen *et al.* [108] identified emotional and pragmatic explanations. Emotional reasons included good support networks (*i.e.*, no need to share their lives with an animal), not wanting another ‘child’, and not wanting to go through the grieving process when the pet dies. Pragmatic reasons included living arrangements (e.g., retirement village which might not allow pets), the desire to travel (and not worry about the care of a pet), health concerns (such as falls, cleanliness of the pet), the expense of keeping a pet, and worries about what would happen to the pet if the older person was no longer able to care for it. This is despite the development of some support programs to help older people keep pets in their lives. One established program is the Royal Society for the Prevention of Cruelty to Animals (RSPCA) *Pets for Older Persons* program, which helps older people by looking after pets during times of personal crisis, such as a medical emergency or requirement of the older person for respite care [83]. The program also can assist with veterinary treatment and pet grooming.

Pets can play a role in the resilience of older people due to their recognized positive effects including companionship, a feeling of purpose and self-identity, and a motivation to engage in daily social activity [109,110]. However, research on the benefits of pet ownership for older people is scant, often anecdotal or methodologically weak, and shows mixed results [108,111]. There may be negative effects relating to pets as trip hazards that increase the likelihood of falls in the older person [112,113].

There is scant evidence in the literature of animal attachment impacting on the behavior of older people during disasters specifically. One US study found that ‘pet ownership was not a significant risk factor for evacuation failure of households with seniors’ [2] (p. 661). They also recognize the other possible evacuation impediments related to old age in general, as noted above.

### 4.5. People with Disabilities

A disability is a long-term physical, mental, intellectual or sensory impairment which intersects with social, political and economic barriers to hinder a person’s full and equal participation in society [114]. Disabilities and activity limitations include: conditions which interfere with mobility (e.g., arthritis, chronic fatigue syndrome), respiratory conditions (e.g., asthma), cognitive difficulties (e.g., dementia), vision loss and hearing loss [115]. In 2009, four million Australians (18.5% of the population) reported having a disability [116].

People with a disability suffer higher rates of mortality and injury in disasters [117]. In relation to preparedness, research has found that whilst people with a disability may have located a shelter or prepared medical supplies, they were overall inadequately prepared for disasters [118,119,120]. There is also a lack of preparedness at an institutional level. For example, Kendall-Tackett & Mona [121] found that the majority of emergency personnel are not trained in responding to people with a disability. Rooney and White [122] note that people with a disability have been left behind in emergency situations because they were not assisted or accommodated within building and emergency plans. In relation to accessing information, current emergency communication networks do not take into account people with a hearing disability and those with vision impairment [123]. In relation to evacuation, most evacuation plans require people to be able to walk, drive, see, or hear and thus are not accessible by people with disabilities [124], which means many cannot evacuate under current systems. There are also specific evacuation shelter needs to take into consideration. Takahashi *et al.* [125] found that people with an intellectual disability were rejected and segregated by their neighbors in refuges due to social discomfort, which created extreme distress for the people with disabilities. Finally, in relation to recovery, loss of supports and aids such as vehicles, medical equipment and accessible housing severely limits independence post-disaster [117].

The existing research on animals as disability aids highlights additional benefits to social, emotional and psychological wellbeing. Lane, Nicholas and Collis [126] surveyed 57 people with a disability assistance dog. They reported an increased sense of social integration and enhancement to self-perceived health. Hart, Zasloff & Benfatto [127] surveyed 38 people with hearing assistance dogs and found that, as well as providing assistance by alerting them to sounds, hearing dogs also increased the owner’s sense of safety, decreased feelings of loneliness especially through changing their interactions within their family and with the hearing community, and also reduced stress. Whitmarsh [128] surveyed 800 visually impaired people. Their respondents cited the benefits of guide dog ownership as: independence, confidence, companionship, increased and changed social interaction and increased mobility. Guest *et al.* [129] undertook a longitudinal study of 51 people with a hearing impairment which measured psychological health and wellbeing prior to receiving a disability assistance dog and up to 18 months after they received their dog. They found that after receiving a dog there were significant reductions in hearing-related problems, as well as in tension, anxiety and depression, and improvements in social involvement and independence. These findings are supported by Valentine *et al.* [42]. There is also research demonstrating the strong bond between people with a disability and their assistance animals [126], and intense grief when the dog dies [130].

### 4.6. The Homeless

There were 105,237 homeless people in Australia in 2011, representing 0.5% of the population [131]. There is scant Australian research on homelessness and disaster preparedness and response [132]. From the existing research, there are four risk factors which increase homeless people’s propensity to death, injury and loss during a disaster and to poor recovery post disaster: (1) lack of resources, (2) lack of access to services, (3) limited social inclusion, and (4) pre-existing physical, mental and emotional stressors [133]. These factors have significant implications for preparedness, communication, transportation, evacuation, sheltering, health and recovery [134]. In particular, people experiencing homelessness have limited or no access to the media through which disaster warnings and information are commonly communicated such as television, radio, and internet. As homeless people have not been included in disaster planning in Australia, they are less likely to be warned, found and evacuated, or provided with adequate support post-disaster [133,135]. Pre-existing trauma heightens the experience of disaster and may mean that homeless people are more vulnerable to post-traumatic stress disorder [134]. In terms of recovery from a disaster, people experiencing homelessness will also be more vulnerable because of the disruption to safe places, sleeping places and income (e.g., Big Issue, recycling, the underground economy) [134]. Moreover, their homelessness may make them ineligible for post-disaster assistance programs, such as housing support [136].

There are no publicly available statistics on the rate of pet or animal ownership amongst people experiencing homelessness in Australia. Rates are likely to be similar to those in the US, where between five and ten percent of people experiencing homelessness also have a pet [137].

Existing research on the relationship between people experiencing homelessness and their pets is primarily qualitative and is US-based. Brewbaker [138] found that dogs were a source of comfort amidst the hardships of homelessness, improving emotional, physical and social wellbeing. Irvine [139] found that dogs helped reduce the social isolation of homeless people by facilitating contact with others. They also provided a ‘moral identity’ both for the person and for others. This is important, given that people experiencing homelessness often internalize many of the dominant negative discourses that they are lazy, unacceptable and irresponsible. Caring for and being responsible for a pet helps homeless people change these internalized stories about themselves. Being seen with a dog also tends to discourage passers-by from yelling abuse [140,141]. Rew [142] interviewed 42 homeless young people, all of whom reported deep feelings of loneliness. Thirteen of the young people reported that in coping with this loneliness, their pets provided unconditional love, reduced loneliness, and improved their health. They also provided companionship and friendship [141]. In fact, Taylor *et al.* [143] found that the attachment bond was stronger between homeless people and their pets than for those who have secure housing [144].

When the bond between person and pet is severed, people experiencing homelessness report significant grief. In an Australian study, Slatter *et al.* [141] interviewed 26 homeless people living in Australia’s Gold Coast about their experiences of pet ownership. Many spoke of grief, loneliness and declining mental health on losing an animal when they became homeless, not being able to keep their pets in shelters, or through not being able to meet council regulations. Being separated from their pets through loss or death is a time of significant grief [141]. In particular, the loss of a pet is one of the most significant stressors for children who are experiencing homelessness [145,146].

This research on the bonds between homeless people and their pets, and the role of pets in reducing isolation and improving mental and physical wellbeing are supported by anecdotal evidence. For example, Munro [147] presents the story of Chris Curran, who felt that people stopped to chat and smile at him more when he was with his dog Princess, rather than ‘looking down on’ him. Many people with whom Munro spoke chose to stay with their pets on the streets rather than enter shelters where their pets were not allowed.

As noted above, research on homeless people and disasters is meager, and research on the role that animals might play is non-existent. However, as with other groups, it is expected that homeless people will be reluctant to leave their animals behind as part of a disaster evacuation. They may also struggle to find evacuation shelters that will accommodate them and their animals, due to a lack of animal vaccination records and other documentation.

### 4.7. People with Mental Health Issues

Mental illness is a general term that encompasses ‘a health problem that significantly affects how a person feels, thinks, behaves and interacts with other people’ [148]. Disorders include affective and anxiety disorders, substance abuse, and psychotic disorders (schizophrenia and bipolar disorder). In 2007, 45% of the Australian population (7.3 million) had experienced an anxiety or affective disorder (e.g., PTSD, depression, bipolar), or a substance abuse disorder at some stage of their lives, and 20% (3.2 million) had experienced them in the previous 12 months [149]. As with people experiencing homelessness, there are four risk factors which increase the vulnerability to death, injury and loss during a disaster and to poor recovery post disaster for person’s experiencing a mental illness: (1) lack of resources, (2) lack of access to services, (3) limited social inclusion, and (4) pre-existing physical, mental and emotional stressors [133].

There are a wide variety of symptoms, treatments, effects and levels of severity of mental illness. People with a panic disorder may struggle to leave the house whereas people with a psychotic disorder may lose touch with reality. The severity can range from mild to severe. Despite this, mental health consumers are no less likely than others to demonstrate heroism, resilience and adaptability to disaster. However, there are some common correlates of mental health issues that may increase their risk in a disaster. These vulnerabilities arise from a lack of resources, services and inclusion that affects their preparedness, communication, transportation and evacuation, sheltering and recovery. For example, people experiencing mental health issues may have fewer social networks. More limited access to the mainstream methods of communicating emergency information can render them unaware of an emergency. They may also be unfamiliar with emergency language and personnel and more likely to react negatively to seeing people in safety clothing, interacting with unknown people, and responding to demands [150]. Post-disaster services may not be appropriate for people with a mental illness [151] and volunteers usually lack appropriate mental health training [150]. There are also particular vulnerabilities relating to pre-existing health conditions. People with a mental illness are more vulnerable to rapid and unplanned changes [151]. A disaster or emergency may trigger the onset of new or recurrent symptoms, or a relapse of substance abuse [152].

There are no comprehensive statistics on pet ownership levels amongst those experiencing a mental health issue in Australia or elsewhere. One study reports that 18.6% of clients of a community mental health service in the US owned a pet, and 63% would like to live with a pet [153]. Other studies examine how pet ownership affects the wellbeing of people with a mental illness. Wisdom *et al.* [154] interviewed 177 adults with a serious mental illness and found that animals assisted recovery through providing empathy and connections which increased social networks, serving as family when these are absent, supporting self-efficacy and a sense of empowerment. In a study of women who had been recently widowed, those who owned pets reported lower drug use and fewer psychiatric symptoms than those who did not own pets [155].

There is also research on the relationship between pet ownership and mental wellbeing more generally [156]. One study of the impact of animal assisted therapy (AAT) on psychiatric patients showed significant decreases in anxiety [157]. AAT decreased violent behavior, and increased language and social skills in children with ADHD [158]. However, responsibility for pet care can exacerbate mental health issues [154].

Further, and relevant for consideration in disaster response, is that higher attachment may be adverse in terms of the effects of the loss of a pet or animal, or the desire/ability to leave them behind [159]. If people with mental health issues do evacuate, they may struggle to find evacuation shelters that will accommodate them due to the stigma attached to their illness, combined with their desire to accommodate their animals.

## 5. Results and Discussion

### 5.1. How do Animals Impact the Disaster Resilience of Vulnerable Groups?

Despite the different causes for vulnerability discussed above, and the different ways in which animals are incorporated into the lives and belief systems of the seven vulnerable groups, it is clear that animals contribute positively to the health and wellbeing of vulnerable people. They provide companionship, security, practical assistance and the social capital that counters loneliness and social isolation.

It is therefore hardly surprising that the ways in which vulnerable people perceive and react to natural disasters is influenced by their interactions and relations with pets and other animals. For vulnerable people, animals can influence:

- Perception and recognition of disasters. For example, assistance animals can help in alerting older people to sirens or other emergency warnings.
- Evacuation decisions. For example, homeless people may refuse shelters if they cannot be accompanied by their animals.
- Evacuation behavior. For example, separation from animals can immobilize owners with disability.
- Evacuation experience. For example, separation from animals can exacerbate the social anxiety of those with mental health issues, whilst the presence of animals in shelters may induce anxiety in some CALD people.
- Recovery from disaster. For example, without hunting dogs, Indigenous Australians’ ability to hunt for food may be compromised. The loss of pets can increase risk of stress disorders in children and social isolation in older people.

With all of these animal-related impacts on disaster behavior in mind, and to preserve the everyday benefits of pets and other animals to vulnerable groups, animals should be accommodated in disaster management and emergency planning at all jurisdictions and across the community-responder divide.

### 5.2. What Role Could Animal Attachment Play in Building the Disaster Resilience of Vulnerable People?

Despite different mechanisms and manifestations of vulnerability amongst the seven groups discussed above, the overarching challenges to building their resilience are the four interrelated areas of access, communication, motivation to act, and recovery. To increase their natural disaster resilience, vulnerable people need information that encourages them to make informed decisions leading to action. However, vulnerable people are often socially isolated and thereby difficult to contact for the purposes of successfully delivering information. They may not have the requisite skills or abilities to fully utilize existing initiatives that are mostly in written form and in the English language. They may also face emergencies on an everyday basis that attune them to their short-term needs, and therefore be less engaged with resilience building initiatives for unpredictable events like disasters. Pets, animals and animal-attachment could be used to overcome these challenges in the following ways.

Animals could facilitate better access to vulnerable people. Information about preparing animals and pets for disaster survival should be standard in general disaster resilience initiatives. But how do we make sure that that information reaches vulnerable people with animals? Disaster resilience building information could be distributed to vulnerable people who have pets by utilizing existing animal-related activities and networks. For people with disabilities, this could be Assistance Dogs Australia. For the homeless, it could be through the RSPCA program *Living Ruff*, the New South Wales program *Pets in the Park*, and the Queensland program *Pawprints*, which provide pet health clinics and free veterinary services, as well as food and emergency shelter for animals.

One means of attracting homeless people into animal-related networks could be via encouraging them to microchip and vaccinate their pet. To create new animal-related networks, these services could be provided free of charge to those who could not otherwise afford them. Microchipping and vaccination could increase acceptance of homeless people and their pets in emergency shelters. Using animals as a conduit for disaster resilience initiatives could have the added benefit of building social capital whilst reducing perceived threat from communicators. This is particularly relevant for those vulnerable groups who experience social isolation and those who may be suspicious or feel threatened by strangers.

Animals could facilitate better communication with vulnerable people. In a study of emergency response for the Sudanese community, Glasgow [160] identifies several engagement strategies that can be entirely independent from written material, such as visual resources, face to face demonstrations, settlement support services, ethnic community groups and ethnic radio stations. Similarly, information in animal-related networks and activities (such as pet care seminars, or free animal health checks) could be used to communicate disaster resilience information. As communication should also be accessible to people with different abilities (especially those with hearing and sight impairment), determining how animal attachment can exactly be used to increase disaster resilience could drive innovation in oral or visual media communication.

Animals could facilitate better motivation for vulnerable people to prepare and act. As described in the introduction in relation to the general population, a desire to protect their pets and animals could motivate vulnerable people who would not otherwise take resilience building actions (be that staying and defending, evacuating with pet or evacuating without pet).

Animals could facilitate better recovery for vulnerable people. Vulnerable people often face additional challenges during recovery from a disaster, due to issues with social and cultural isolation, mental health, developmental stresses (in the case of children) and age-related conditions. As pets and other animals provide assistance for these challenges in daily life, recovery without pets can lead to their exacerbation in life after a disaster. During disasters, owners and responders may be faced with the sight of distressed or dying animals, and sometimes the task of euthanizing them. This adds another layer of emotional distress to recovery (see, for example [23]). Avoiding these disturbing experiences, and maximizing the value of pets and other animals in improving the recovery of vulnerable people after disasters is a compelling rationale for ensuring that all measures are taken to ensure that pets as well as people survive natural disasters. As animal lives are so frequently dependent on humans for survival, sometimes animals are reliant on the actions made by (or available to) vulnerable people. The benefits of animals to vulnerable people during disaster recovery and rebuilding are not confined to animal guardians alone. Animal assisted therapy and simple animal-assisted interactions can facilitate and improve interpersonal interactions.

### 5.3. Recommendations for Further Research

Further empirical research is required to further substantiate the literature and theory based proposition made in this article that animal attachment, activities and networks can be used as conduits for building disaster resilience amongst vulnerable people. The following avenues for research would be valuable:

(1) Systematic collection of data on pet and animal ownership (by the Australian Bureau of Statistics (ABS) or Australian Companion Animal Council (ACAC)) that can indicate or provide:
  - The vulnerability of respondents.
  - Incidence and prevalence of pet and animal owners.
  - The numbers and types of pets and animals owned.
  - Language spoken at home.
(2) Forecasting of pet and animal ownership trends, especially for CALD groups.
(3) An understanding of the impact of non-domestic/non-household animals (*i.e.*, birds, horses, donkeys, alpacas) on vulnerable people
(4 A social network analysis to comprehensively understand animal-related social networks, including their reach and media and communication channels
(5) A study of the most effective ways of using animal attachment and animal-related activities and networks to promote disaster resilience (media communication, *etc.*)
(6) A study of the most effective ways for vulnerable people to evacuate with their animals (e.g., interactions with responders)
(7) Practical translation of plans for vulnerable people to evacuate with their animals for the purposes of identifying barriers to best practice and areas requiring further information and assistance (such as walkthroughs)
(8) An understanding of the role of pets and animals in rebuilding lives and communities after a disaster, with a focus on vulnerable groups or a consideration of vulnerability as it applies to all those who have survived disasters
(9) Other vulnerable groups, such as:
  - People who live alone or without ‘personal or community support networks to help them in an emergency’ (who are likely to meet the criteria for Vulnerable Persons Registers) [40] (pp. 4–5).
  - Indigenous Australians living in non-remote communities.
  - Non-indigenous Australians living in remote communities.
  - People who have previously survived a disaster.
  - Animals (and how humans impact their disaster resilience, building on Irvine’s [21] research).

The research proposed above could (a) support further evaluation for the case for using animal attachment to increase the disaster resilience of vulnerable people and (b), identify the best means of doing so (what works best for whom and under what circumstances).

## 6. Conclusion

This article critically evaluated the proposition that animal attachment could be used to build disaster resilience [3] even for vulnerable groups. It identified the importance of pets and other animals in the lives of vulnerable people as well as their potential contribution to disaster resilience. In particular, animal attachment and animal related activities and networks could be useful conduits for successfully accessing vulnerable people, communicating resilience building information, engaging pet and animal owners and guardians in resilience building behaviors and facilitating recovery. Of course, not all vulnerable people own animals. However, ownership is high in developed countries and those who do not own animals still have animals in their lives or in their social networks. Moreover, this animal-related ‘piggybacking’ proposition may have analogies to other situations where people (more or less vulnerable) experience Attachment or what Belk [19] refers to as a relationship of ‘extended self’ such as houses [161]. Animal attachment and animal-related activities and social networks may provide novel and innovative means to improve disaster preparedness and responsiveness, but the success of the kinds of initiatives suggested throughout this paper may be impeded by pragmatic limitations. For example, it has been incredibly difficult to educate non-vulnerable populations in the US about animal friendly shelters during evacuations [162]. Notwithstanding the challenges to any public health initiative such as funding and government support, there is a clear need to identify who is most at risk of natural disasters and determine how best to access and engage them. However, protection is rarely if ever absolute. We are all vulnerable people and we are all non-human animals. Research and planning should therefore aspire to enable and motivate maximum disaster resilience for all members of the community—humans and nonhuman animals alike; pets and their people.
